# Flexible Memristive Organic Solar Cell Using Multilayer 2D Titanium Carbide MXene Electrodes

**DOI:** 10.1002/advs.202300433

**Published:** 2023-05-03

**Authors:** Kiran A. Nirmal, Wanqi Ren, Atul C. Khot, Dae Yun Kang, Tukaram D. Dongale, Tae Geun Kim

**Affiliations:** ^1^ School of Electrical Engineering Korea University Anam‐ro 145, Seongbuk‐gu Seoul 02841 South Korea; ^2^ Computational Electronics and Nanoscience Research Laboratory School of Nanoscience and Biotechnology Shivaji University Kolhapur 416004 India

**Keywords:** flexible organic solar cells, flexible transparent electrodes, memristors, neuromorphic computing, Ti_3_C_2_T*_x_* MXene

## Abstract

Hybrid systems have attracted significant attention within the scientific community due to their multifunctionality, which has resulted in increasing demands for wearable electronics, green energy, and miniaturization. Furthermore, MXenes are promising two‐dimensional materials that have been applied in various areas due to their unique properties. Herein, a flexible, transparent, and conductive electrode (FTCE) based on a multilayer hybrid MXene/Ag/MXene structure that can be applied to realize an inverted organic solar cell (OSC) with memory and learning functionalities is reported. This optimized FTCE exhibits high transmittance (84%), low sheet resistance (9.7 Ω sq^−1^), and reliable operation (even after 2000 bending cycles). Moreover, the OSC using this FTCE achieves a power conversion efficiency of 13.86% and sustained photovoltaic performance, even after hundreds of switching cycles. The fabricated memristive OSC (MemOSC) device also exhibits reliable resistive switching behavior at low operating voltages of 0.60 and −0.33 V (similar to biological synapses), an excellent ON/OFF ratio (10^3^), stable endurance performance (4 × 10^3^), and memory retention properties (10^4^ s). Moreover, the MemOSC device can mimic synaptic functionalities on a biological time scale. Thus, MXene can potentially be used as an electrode for highly efficient OSCs with memristive functions for future intelligent solar cell modules.

## Graphical abstract

A highly flexible, transparent, and conductive electrode is fabricated based on a multilayer hybrid MXene/Ag/MXene structure, which is then utilized for realizing an organic solar cell (OSC) with memory and learning functionalities. The fabricated device (memristive OSC) exhibits a power conversion efficiency of 13.86%, along with excellent nonvolatile resistive switching properties and synaptic learning functionalities at biological voltages and time scales.

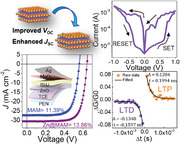

## 1 Introduction

With the development of increasingly sophisticated consumer electronics, portable and wearable power sources are in demand for modern applications. Accordingly, flexible organic solar cells (OSCs) have gained significant attention due to their various outstanding qualities, including low cost, low weight, ease of fabrication, mechanical flexibility, and notable power conversion efficiency (PCE).^[^ ^1^, ^2^ ^]^ Recently, the emerging framework of the internet of things (IoT) has become popular in various industrial sectors and within consumer electronics to satisfy the needs of different stakeholders. However, although IoT edge devices have gained increasing importance, they rely on external power sources (such as batteries). To address this issue, IoT devices with self‐energy harvesting through solar cells is preferred with advantages of no need to replace batteries, increased device lifetime, and further miniaturization. In addition, neuromorphic computing through a simple two‐terminal memristor structure is an ideal solution for IoT‐oriented energy‐efficient computing, pattern recognition, and visual preprocessing, exhibiting superiority over the von Neumann computing architecture.

Recently, two‐terminal memristors have gained the attention of the scientific community because they act as single synaptic units with high scalability, fast switching speeds, and 3D integration capability.^[^ ^3^ ^]^ Moreover, the operating mechanisms of these organic materials are consistent with human physiology and their mechanical properties match those of tissue.^[^ ^4^ ^]^ However, exploiting the interesting nature of organic materials and designing and implementing single nanodevices with multifunctionalities remain challenging. Conventional self‐powered memory devices consist of two independent units connected by wires: a solar cell and a memristor.^[^ ^5^ ^]^ Although these systems can achieve simultaneous power generation, data storage, and processing, they face some technical challenges, such as being bulky, inflexible, and expensive. More importantly, they suffer from energy losses through the externally‐connected wires. These drawbacks could be overcome by integrating a memristor and a solar cell into a single unit. A perovskite photovoltaic stack for parallel energy harvesting and memory computing has been reported previously. For instance, Rogdakis et al. reported a memristive perovskite solar cell with ≈17% PCE and a 10^5^ switching memory window. In addition, the cell demonstrated stable endurance and retention of 3 × 10^3^ cycles and 3600 s, with basic synaptic functionalities.^[^ ^3^ ^]^ Similarly, Loizos et al. deployed a wide variety of synaptic properties of a four‐cation RbCsFAMA perovskite device across an inverted solar cell geometry.^[^ ^6^ ^]^ In addition, a multifunctional device based on n‐perovskite/p‐spiro‐MeOTAD p‐n heterojunction diode that enables the integration of photovoltaic, photodetection, and synaptic functionalities in a single cell has been demonstrated.^[^ ^7^ ^]^ Notably, the functional integration of a ferroelectric oxide thin film and an organic bulk heterojunction (BHJ) to achieve a transistor effect has been reported.^[^ ^8^ ^]^

On the other hand, the different optoelectronic and physical properties of transparent electrodes deposited on glass and plastic substrates are responsible for the excellent performance of OSCs. Hence, highly transparent, mechanically stable, and low sheet resistance flexible transparent conducting electrodes (FTCEs) are a key component when designing efficient solar cells, since they play a decisive role in device performance.^[^ ^9^ ^]^ Indium tin oxide (ITO) is currently used commercially as a transparent electrode due to its high optical transparency (>80% in the visible region) and low resistivity (≈10–20 Ω sq^−1^). However, ITO suffers from several critical drawbacks, including poor mechanical properties (such as brittleness), high cost, and low conductivity on flexible substrates.^[^ ^10^ ^]^ To date, several materials have been extensively investigated as promising alternatives for ITO, such as carbon nanotubes, graphene, transparent conducting polymers, metal meshes, and metal nanowires.^[^ ^11^, ^12^, ^13^, ^14^, ^15^ ^]^ Although the use of these electrodes facilitated significant improvements in the performance of solar cells, several technical issues, including insufficient electrical conductivity, critical fabrication protocols, and limited work function (WF), remained key challenges when designing direct integrating and highly scalable photovoltaics.

Among the class of two‐dimensional materials, newly emerged transition metal carbides, nitrides, or carbonitriles (popularly known as MXenes) are excellent electronic materials with very high metallic conductivity and excellent mechanical properties. Moreover, their super hydrophilicity provides an excellent platform for solution processing approaches. Common wet chemical protocols for the synthesis of MXenes introduce surface terminal groups (including –OH, –O, –Cl, and –F) that control the WF of MXenes and change the WF from 1.6 to 6.0 eV.^[^ ^16^ ^]^ Although MXenes have high conductivity, they still lag behind metal‐based electrodes.^[^ ^17^ ^]^ In addition, fabricating MXene‐based FTCEs with sufficiently low sheet resistance is challenging due to their numerous grain boundaries,^[^ ^18^ ^]^ impurities,^[^ ^19^ ^]^ and structural defects.^[^ ^20^ ^]^ Although FTCEs using MXene and its hybrid structure have been explored previously, nonuniform coverage with Ag nanowire and MXene can diminish their performance.^[^ ^16^, ^21^ ^]^ Importantly, promising advances in MXene‐based FTCEs have not yet been reported, and the outstanding photoconversion efficiency of OSCs using MXene and its hybrid structures remains a critical challenge.

Accordingly, in this paper, we demonstrate a multifunctional device based on organic BHJ and MXene FTCE that enables photovoltaics and memristive functions to be integrated within a single device. We believe that integrating the solar cell and information processing elements can result in the emergence of intelligent solar modules that can track and respond to changes in the angle of sunlight.

In this study, we exploited surface‐modulated Ti_3_C_2_T*_x_* MXene/Ag/MXene stacked films as an FTCE and used a one‐step‐solution processible technique to modify the MXene surface using Ag, Ni, and Zn nanoparticles (NPs) to tune its WF. Spin coating and sputtering techniques were used to obtain MXene electrode films with a low sheet resistance of 9.7 Ω sq^−1^ g, high optical transmittance, and excellent mechanical stability. The effectiveness of our strategy for engineering the surface was demonstrated by employing an MXene/Ag/MXene multilayer configuration as FTCEs for a proposed memristive OSC (MemOSC). The Zn‐modulated MXene‐based OSCs exhibited a high PCE of 13.86%. Moreover, the same organic BHJ with MXene FTCE demonstrated reliable memristive switching for the emulation of biological synaptic functionalities. Thus, the successful integration of an OSC and a memristor synaptic element can result in the production of intelligent solar cell modules.

## 2 Results and Discussion

### 2.1 Crystal Structure and Morphologies

**Scheme 1:**
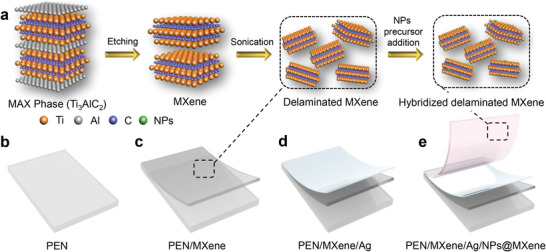
a) Synthesis process of liquid exfoliation and delamination utilized for fabrication of a multilayer electrode. Step‐by‐step protocol for the fabrication of multilayer electrode: b) oxygen plasma treated PEN substrate, c) MXene deposition by spin coating, d) sputtered deposited thin layer of Ag, and e) multilayer PEN/Ti_3_C_2_T*_x_* MXene/Ag/NPs@Ti_3_C_2_T*_x_* MXene electrode.

Given the remarkable electronic conductivity and WF tunability of Ti_3_C_2_T*_x_* MXene, it is an excellent candidate to be used as a transparent electrode. We synthesized Ti_3_C_2_T*_x_* and its hybrids through a selective etching technique, which is schematically portrayed in **Scheme** **1** and is described in the experimental section (Supporting Information). Well‐ordered two‐dimensional Ti_3_C_2_T*_x_* sheets were detected in the scanning electron microscopy (SEM) image (Figure S1a, Supporting Information) and Al was absent in energy dispersive X‐ray spectrometry (EDX) mapping (Figure S1b, Supporting Information), confirming complete etching of the Ti_3_AlC_2_ MAX phase. We further delaminated etched powder to form colloidal solutions of Ti_3_C_2_T*_x_* (DMX) (Figure S2a, Supporting Information) and its composites using a mild exfoliation protocol and soft chemical processing. The oxidation of MXene under high‐temperature and humid environments can be prevented by introducing positive metal ions on the MXene nanosheets because metal ions readily bond with oxygen‐containing groups. Compared to Ag and Ni, Zn has higher reactivity,^[^ ^22^ ^]^ less deformability, better flexibility, and higher transparency.^[^ ^23^, ^24^ ^]^ To utilize Ti_3_C_2_T*_x_* MXene as an FTCE, we sandwiched a thin Ag layer between the MXene layers and achieved a window of WF ranging from 4.61 to 5.08 eV, which provided positive and negative changes in the WF of the MXene. We also introduced the thin Ag layer to achieve 100% coverage, avoid any adverse effects on the transparency, increase metallic conductivity, and fill any surface voids. Moreover, the functional groups of MXene had a strong interaction with the polyethylene naphthalate (PEN) substrate, enhancing surface adhesion.

**Figure 1:**
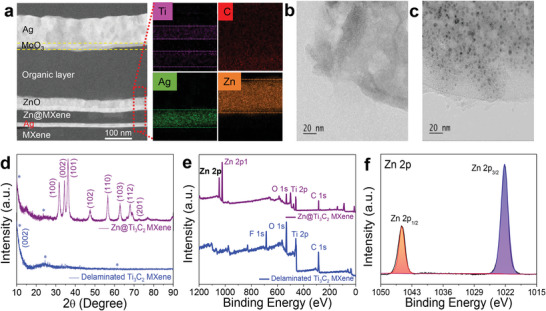
a) High‐resolution cross‐sectional TEM image of OSC fabricated using Zn@Ti_3_C_2_T*_x_* MXene electrode and the corresponding TEM assisted EDX element mapping of Zn@Ti_3_C_2_T*_x_* MXene electrode. High‐resolution TEM images of b) pristine MXene and c) hybrid Zn MXene. d) XRD patterns, and e) XPS survey spectra of pristine MXene and Zn@Ti_3_C_2_T*_x_* MXene. f) High‐resolution core level spectrum of Zn 2p.

**Figure** **1**a presents a cross‐sectional transmission electron microscope (TEM) image of the proposed multilayer Zn@DMX/Ag/DMX (Zn@MAM) electrode for OSC applications. The high‐resolution TEM (HRTEM) image of the OSC distinguished the individual layers effectively. For a detailed analysis of the electrode, we employed TEM‐assisted EDX elemental mapping to identify the uniform distribution of the required elements. The major challenge in solar cells is to design electrically connected interlayers that are physically separated.^[^ ^25^ ^]^ Sharp interfaces of the individual layers indicate that there is no mixing of layers or physical damage to the device. A type of delamination was observed in the middle of the ZnO layer, which could have been caused by the low sputtering power (40 W) and temperature used in this study. This issue could be resolved by further optimizing the deposition conditions (if required).^[^ ^26^, ^27^ ^]^ Additionally, the EDX mapping images of the Ti, C, Ag, and Zn suggested that Zn was uniformly doped throughout the top Zn@Ti_3_C_2_T*_x_* MXene layer of the FTCE.

The morphology of delaminated Ti_3_C_2_T*_x_* MXene and its hybrids were also analyzed using TEM. The resulting colloidal solution of Ti_3_C_2_T*_x_* and its hybrids contained nanosheets consisting of two carbon atoms bounded to three titanium atoms as an elementary unit. Figure 1b,c display a single sheet of DMX and Zn hybrid Ti_3_C_2_T*_x_* DMX (Zn@DMX), respectively. The TEM images confirmed the presence of Ag and Ni hybrid DMX, as depicted in Figure S3a,b, Supporting Information, respectively. The HRTEM image of pristine DMX exhibited a layered feature of MXene with a spacing of 0.32 nm (Figure S3c, Supporting Information). Moreover, the crystallinity of the Zn NPs (Zn@DMX) was supported by lattice fringes with a spacing of 0.19 nm (Figure S3d, Supporting Information). The phase transitions of hybridization were confirmed using the X‐Ray diffraction (XRD) technique. The broad peak (002) of DMX in Figure 1d was attributed to the delamination of the Ti_3_C_2_T*_x_* sheets. The pattern of Zn@DMX revealed mixed peaks that originated from the Zn and the Ti_3_C_2_T*_x_* structures, confirming the successful synthesis of the hybrid structure. Four diffraction peaks of Ag appeared at 38.07°, 44.25°, 64.5°, and 77.3° (Figure S4a, Supporting Information), indicating the successful formation of the Ag@Ti_3_C_2_T*_x_* MXene (Ag@DMX) hybrid. Moreover, diffraction peaks of Ni@Ti_3_C_2_T*_x_* MXene (Ni@DMX) were observed at 44.4°, 51.9°, and 76.4°, which were related to the (111), (200), and (220) planes of Ni, respectively. A large reduction in the (002) peak of Ti_3_C_2_T*_x_* was also observed, which was caused by the excellent coverage of NPs.

### 2.2 Bonding States, Work Function, and Optical Properties

The functional groups, electronic structure, and composition of Ti_3_C_2_T*_x_* MXene were evaluated using X‐ray photoelectron spectroscopy (XPS). The survey spectrum indicated the presence of Zn (Figure 1e), Ag, and Ni (Figure S4b, Supporting Information) peaks in addition to DMX, which confirmed the formation of hybrids. Two main peaks of Zn@DMX (i.e., Zn 2p_1/2_ and Zn 2p_3/2_) appeared at 1045.6 and 1022.2 eV (Figure 1f), respectively, suggesting the presence of Zn^2+^ in a hybrid structure. Moreover, the core‐level Ni 2p spectra of Ni@DMX (Figure S4c) exhibited a 2P_3/2_ peak, a major peak at 856.0 eV (Ni^2+^), and a shoulder satellite peak at 862.1 eV. Similarly, 2P_1/2_ presented three peaks at 871.1, 874.2, and 880.5 eV, which corresponded to Ni^2+^, Ni^3+^, and the satellite peak, respectively. Based on the XPS analysis, ultra‐small nickel NPs confirmed the high number of reactive oxidation states (such as Ni^2+^ and Ni^3+^), which could encourage the adsorption of O_2_ on the MXene sheets via the coverage of NPs, which would reduce electrode performance. The Ag 3d core‐level spectrum of the Ag@DMX hybrid is displayed in Figure S4d, Supporting Information. Here, two characteristic peaks of silver located at 368.2 and 374.2 eV were assigned to Ag 3d_5/2_ and Ag 3d_3/2_, respectively, indicating the self‐reduction of silver. The spin energy separation between the 3d doublet of Ag was 6.0 eV, suggesting the formation of metallic AgNPs on the MXene nanosheets. The XPS details of the different hybrids are provided in Figure S5, Supporting Information, and the SI file. Scheme 1b–e presents the step‐by‐step protocol employed for the fabrication of the multilayer electrodes. Typically, this process involved coating the PEN substrates with MXene, after which a thin layer of Ag was sputter deposited to obtain high metallic conductivity and fill the surface voids. Subsequently, hybrid MXene colloidal solutions of DMX and NPs were spin‐coated to fabricate DMX/Ag/DMX (MAM), Ni@DMX/Ag/DMX (Ni@MAM), Ag@DMX/Ag/DMX (Ag@MAM), and Zn@MAM multilayer structures (Figure S2b, Supporting Information).

**Figure 2:**
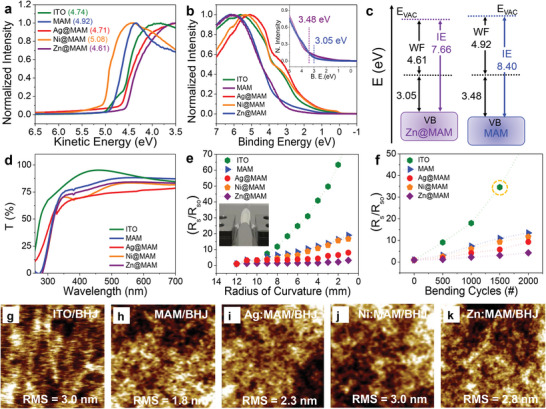
a) UPS spectra measured at 21.22 eV around the secondary electron cut‐off. b) UPS spectra displaying the valence band maxima. Inset displays a magnified valence band maximum of Zn@MAM and MAM electrodes. c) Energy level diagram of pristine MXene and Zn@MXene‐based electrodes. d) Optical transmittance of different substrates and electrodes. Mechanical stability of electrodes with respect to the e) radius of curvature and f) bending cycles. AFM images organic BHJ deposited on g) ITO, h) MAM, i) Ni@MAM, j) Ag@MAM, and k) Zn@MAM electrodes.

In practical applications, the critical parameters for electrodes are high transparency and excellent electrical performance. Accordingly, the electronic properties of the fabricated electrodes based on Ti_3_C_2_T*_x_* MXene and its hybrids were evaluated using ultraviolet photoelectron spectroscopy (UPS), which are presented in **Figure** **2**a. The WF of pristine MAM was revealed as 4.92 eV, which was based on the secondary electron onset. The hybridization of MXene with Zn, Ag, and Ni provided an outstanding approach for tuning the WF window from 4.61 to 5.08 eV, as depicted in Figure 2a. Therefore, the proposed strategy was able to tune the WF of MXene and agreed with the XPS results. The spectra measured in the valence band region (Figure 2b) indicated that the intensity elongated up to the Fermi level. Moreover, the formation of a sharp enhancement was spotted at energy values of 3.05 and 3.48 eV for MAM and Zn@MAM, respectively, as displayed in the inset of Figure 2b. The energy level diagram in Figure 2c schematically illustrates WF and ionization energy reduction upon the hybridization of the MXene surface with Zn NPs. The FTCE exhibited excellent alignment of the energy levels with the organic BHJ materials. The small energy offset between the FTCE WF and electron transport layer (ETL) ZnO could effectively enhance photovoltaic performance by reducing energy losses in the device. The UPS valence band spectra and energy level diagrams of ITO, Ag@MAM, and Ni@MAM are depicted in Figure S6, Supporting Information. The overall transmittance spectra (Figure 2d) revealed that the optical transmittance of the FTCEs decreased with the hybridization of MXene. The ITO exhibited a transmittance of ≈91% at 550 nm, compared to ≈87% for MAM FTCE. The electrodes modified by metal NPs demonstrated low and stable sheet resistance. The obtained highest transmittance values at 550 nm for Zn@MAM, Ag@MAM, and Ni@MAM were 84%, 83%, and 75%, respectively, which was caused by the reflection of photons due to the NPs.

### 2.3 Mechanical Stability and Surface Topography of MXene FTCEs

The electrode's mechanical robustness is the most important aspect of flexible solar cells and is evaluated by estimating changes in R_s_ from its elementary value (*R*_sh0_). The bending radius (2–12 mm)‐dependent change in *R*_s_ after the flexing test is displayed in Figure 2e. As previously reported,^[^ ^28^ ^]^ the R_sh_ value of the reference ITO FTCE increased significantly as the radius of curvature decreased. Herein, a modest change in the *R*_sh_ values was noticed for Zn@MAM TCEs, even when bending at different radii of curvature. For the other MXene‐based electrodes, a slight enhancement in *R*_sh_ was observed with decreasing bending radius. We further monitored the mechanical deformation at a fixed radius of 4 mm with up to 2000 bending cycles. Here, a similar behavior was observed in the sheet resistance of the electrodes, and their *R*_sh_ values continuously increased with several bending cycles, as displayed in Figure 2f. The resistance of the Zn@MAM electrode changed negligibly with bending cycles, whereas substantial changes were evident in the ITO and other MXene‐based electrodes. This was because of the high stability and uniformity of Zn compared to the Ag and Ni NPs on the MXene surfaces. We also confirmed that the Zn@MAM multilayer electrode was more favorable than other electrode configurations, providing the device with improved mechanical stability. Subsequently, the impact of the fabricated electrode morphology on the surface topography of the BHJ was investigated using atomic force microscopy (AFM). Figure 2g–k displays AFM images of BHJ deposited on ITO, MAM, Ag@MAM, Ni@MAM, and Zn@MAM, respectively. The designed multilayer electrodes exhibited similar surface topographies, with RMS values ranging from 1.8 to 3.0 nm, which were similar to (or smoother than) ITO (3 nm). The RMS value for Ni@MAM was identified as high, which could have been due to the large particle size of Ni compared to Ag and Zn, as evidenced in the TEM images.

### 2.4 Photovoltaic Performance of OSCs with MXene FTCEs

**Figure 3:**
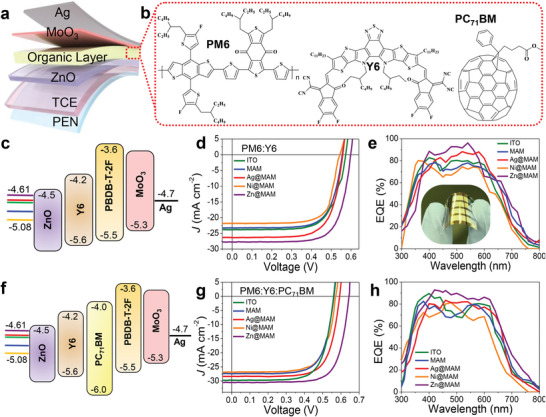
a) Schematic structure of an inverted organic solar cell. b) Chemical structures of organic layers: PM6, Y6, and PC_71_BM. c) Energy level diagram of binary devices based on PM6:Y6 and the corresponding d) *J–V* curves and e) EQE. f) Energy level diagram of the ternary organic layer‐based device and its corresponding g) *J–V* curves and h) EQE.

To demonstrate the suitability and effectiveness of MXene‐based FTCEs in OSCs, they were incorporated into devices with the following configurations: PEN/MXene (30 nm)/Ag (6 nm)/NPs@MXene (25 nm)/ZnO (40 nm)/PM6:Y6:PC_71_BM (140 nm)/MoO_3_ (5 nm)/Ag (120 nm). **Figure** **3**a displays the structure of an inverted OSC device, where PBDB‐T‐2F:Y6 (binary) and PBDB‐T‐2F:Y6:PC_71_BM (ternary, Figure 3b) materials were used as photoactive layers. Commercial viability and high efficiency are crucial factors of solar cells, in addition to manufacturing costs and stability. We studied device performance by incorporating a third element in the organic blend, as this can improve device efficiency and stability.^[^ ^29^ ^]^ Accordingly, OSCs with MAM, Ag@MAM, Ni@MAM, and Zn@MAM were fabricated, and their results are presented in **Table** **1**. An ITO on PET with a resistance of 15 Ω and a transmittance of 91% at 550 nm was used as the reference FTCE. Energy level diagrams of the binary and ternary blends are displayed in Figure 3c,f, respectively. The *J–V* characteristics of best‐performing OSC devices under the illumination of 1 sun (100 mW cm^−2^, AM 1.5 G) using PBDB‐T‐2F:Y6 are presented in Figure 3d. Specifically, the PBDB‐T‐2F:Y6: PC_71_BM‐based cells exhibited maximum efficiency (Figure 3g). Compared to the OSC with pristine MXene‐based MAM electrodes, the Zn@MAM‐doped OSC demonstrated a substantial increase in PCE (up to 13.86%) due to the strong doping effect.

**Table 1:** Detailed photovoltaic parameters extracted from typical *J–V* curves of flexible OPV for various electrodes

Organic blend	FTCEs	*V*_oc_ [mV]	*J*_sc_ [mA cm^−2^]	FF (%)	PCE [%]
Binary	ITO	556 ± 3	23.83	70.85	9.39
	MAM	556 ± 4	23.24	71.13	9.19
	Ni@MAM	544 ± 1	21.82	71.13	8.44
	Ag@MAM	558 ± 3	26.31	70.95	10.42
	Zn@MAM	600 ± 2	27.72	71.23	11.78
Ternary	ITO	561 ± 2	29.86	71.12	11.92
	MAM	561 ± 3	28.41	71.47	11.39
	Ni@MAM	556 ± 1	26.82	71.02	10.60
	Ag@MAM	590 ± 3	28.40	71.14	11.92
	Zn@MAM	641 ± 4	33.49	71.38	13.86

Table S1, Supporting Information, lists the performance of the OSCs according to the employed Mxene. To the best of our knowledge, this is the first study to report on highly efficient OSCs based on MXene electrodes. The small energy offset between the electrode WF and ETL WF significantly reduced energy losses in the device. Relatively low PCEs of 11.92% and 11.39% with low *J*_sc_ values of 28.40 and 28.41 mA cm^−2^ were observed for the Ag@MAM and MAM electrodes, respectively, which were comparable to the results for the ITO‐based device. In contrast, PCE and *J_sc_* clearly reduced to 10.60% and 26.82 mA cm^−2^, respectively, when assembled using a high WF‐based Ni@MAM electrode. The photoresponse in the entire absorption region was examined by measuring the external quantum efficiency (EQE). Figure 3e,h displays the EQE spectra of the binary and ternary blend‐based cells, respectively. A higher photocurrent was obtained with the Zn@MAM‐based device compared to the other devices, which was due to its high transmittance profile and improved conductivity.

### 2.5 Resistive Switching Characteristics of Flexible MemOSC Devices and Correlation Between Photovoltaic and Memristive Performance

**Figure 4:**
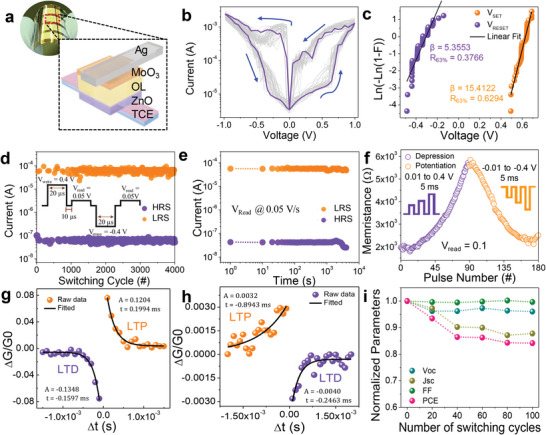
a) Structure of MemOSC device. Memristive b) *I–V* switching, c) Weibull distribution plot of switching voltages, d) endurance, and e) retention of the MemOSC device. Here, *β* and X_63%_ are the shape and scale parameters of the Weibull distribution. f) Potentiation and depression characteristics and STDP based g) anti‐symmetric Hebbian (ASH) and h) anti‐symmetric anti‐Hebbian (ASAH) learning rules mimicked by MemOSC device. Values of the scaling factor (A) and time constant are shown in the inset of ASH and ASAH‐related figures. i) Correlation between OSC performance parameters and the number of switching cycles of the MemOSC device.

Unavoidable charge traps in the active layer with the motion and accumulation of ions are responsible for switchable photovoltaics or memristive behavior. Thus, we utilized this effect for data storage and processing. **Figure** **4**a displays a schematic illustration of a flexible MemOSC. The electrical measurements of Ag/MoO_3_/OL/ZnO/FTCE/PEN were recorded by grounding FTCEs as the bottom electrode (BE) and applying a voltage to the Ag top electrode (TE). Figure 4b presents the typical current‐voltage (*I–V*) curves of a MemOSC device using the Zn@MAM electrode. The resistive switching (RS) effect in the OSC devices was achieved by applying a small voltage. The device exhibited a forming‐free operation, which was attributed to the large number of innate charge carriers in the RS layer. In addition, Ag TE (used as an active electrode) was easily oxidized (Ag^+^ ions) by the application of external biases and contributed to conduction by migrating towards the BE. Furthermore, the presence of MoO_3_ and ZnO also initiated some oxygen vacancies, which facilitated the formation of conductive filaments. Finally, the continuous electric field action on the organic films increased the number of charge carriers. These factors eventually resulted in a permeation network that served as an effective carrier path. Therefore, the device worked effectively without any forming operation. Stable bipolar RS loops were obtained with a high ON/OFF ratio of 10^3^, which was achieved by avoiding the insertion of electron transport and hole transport layers.^[^ ^30^ ^]^ The MemOSC device worked on very low SET and RESET voltages, which were close to the biological voltage scale.^[^ ^31^ ^]^ The box plot distribution, the cumulative probability of switching voltages, and different statistical measures are presented in Figure S7a,b, Supporting Information.

The Weibull distribution plot of the switching voltages suggested that the SET and RESET voltages of the device had reasonably good uniformity due to the higher value of the shape parameter (*β*), as shown in Figure 4c. However, the switching uniformity of the fabricated devices can be improved by introducing a metallic or suboxide layer,^[^ ^32^ ^]^ interfacial triggering,^[^ ^33^ ^]^ or metal doping at the interface.^[^ ^34^ ^]^ The switching speed of the MemOSC was investigated by applying an AC pulse to the device, as depicted in Figure S8, Supporting Information. The delay time was determined as ≈200 ns, which was sufficient for nonvolatile memory applications. The *I–V* characteristics of the flexible MemOSC device under different bending conditions and bending cycles (with a 6 mm bending radius) were also investigated, as shown in Figure S9a,b, respectively. The *I–V* curves indicated that there was no obvious change in switching behavior under different bending conditions and bending cycles, suggesting that the device had good flexibility. Furthermore, the RS characteristics of the MemOSC device were investigated under both visible light and dark environments, as shown in Figure S10a,b, respectively. Although similar switching behaviors were observed for both samples, the SET voltage increased slightly (from 0.54 to 0.71 V) under illumination for 240 s. This increase could have been caused by conformational reconfiguration in the organic structure (similar to photo‐induced trans‐to‐cis isomerization), which depreciated the charge carrier transport upon photoirradiation.^[^ ^35^ ^]^ For reference, light illumination induces a surface potential shift in organic materials, causing a large potential barrier between the electrode and active materials, resulting in a higher SET voltage and lower OFF current.^[^ ^36^ ^]^ Moreover, the amount of the current in the RS device was reduced under light illumination because the photoinduced carriers recombined with the oxygen vacancies, increasing the resistance of the device. In other words, light‐induced carriers prevent the transition from HRS to LRS (SET voltage increase) while assisting the transition from LRS to HRS. The fabricated MemOSC demonstrated reliable operation over 4000 switching cycles by maintaining a high ON/OFF ratio without any observable degradation (Figure 4d), with long memory retention of over 10^4^ s (Figure 4e). A schematic of pulse waveforms used for endurance test is displayed in the inset of Figure 4d, and the constant voltage stress was applied over time for retention test. These results indicated that the MemOSC is a highly reliable nonvolatile memory device. Moreover, the charge transport results suggested that the space charge limited conduction and trap‐assisted tunneling conduction mechanisms governed the conduction of the MemOSC device (Figures S11–S13, Supporting Information).

The gradual change in memresistance noticed under positive and negative bias could be utilized to mimic different synaptic functionalities. Moreover, the obtained analog *I–V* characteristics of the MemOSC device met the basic requirement for synaptic emulation. We attempted to mimic some basic and advanced synaptic learning properties to demonstrate the suitability of the proposed MemOSC device for neuromorphic computing applications. Initially, the potentiation and depression synaptic learning properties were mimicked using a MemOSC device. To achieve this, 90 positive and negative pulses were applied to the MemOSC device and the corresponding changes in memristance (synaptic weights) were recorded to achieve the emulation (Figure 4f). The pulse measurement scheme for the emulation of potentiation and depression is displayed in the inset of Figure 4f. To emulate synaptic functionalities more effectively and avoid the switching layer breakdown, we applied a neural pulse scheme smaller than the average SET voltage (+0.6 V) and RESET voltage (−0.4 V) obtained from multiple *I–V* cycle measurements of the fabricated MemOSC device. The memristance was decreased (or increased) as the potentiation spikes (or depression spikes) were applied to the device. These results suggested that the proposed MemOSC device mimicked the basic synaptic learning properties fairly accurately. Furthermore, modifying the synaptic weights of the MemOSC device could be utilized by mimicking advanced spike timing‐dependent plasticity (STDP) rules.

Figure S14, Supporting Information, displays the pulse measurement schemes of the STDP learning rules. In neuroscience, Hebbian learning‐based STDP rules are most important and can be mimicked by adjusting the time intervals between the pre‐ and post‐synaptic spikes.^[^ ^37^ ^]^ Accordingly, we mimicked two important STDP learning rules (anti‐symmetric Hebbian (ASH) and anti‐symmetric anti‐Hebbian), as displayed in Figure 4g,h, respectively. Based on the ASH Hebbian rule, the device conduction decreased if the pre‐spike preceded the post‐spike (Δt > 0) and resulted in a reinforcement of the synapse strength, which is termed long‐term potentiation (LTP). In comparison, the synapse strength decreased if the post‐spike preceded the pre‐spike (Δ*t* < 0), which is referred to as long‐term depression (LTD).^[^ ^38^ ^]^ In this study, we obeyed the same protocol for stimulating the MemOSC device and successfully mimicked the LTP and LTD characteristics of both STDP learning rules. In computational neuroscience, these types of STDP rules can be fitted with the following exponential function^[^ ^39^ ^]^:

(1) $$\Delta W\;=\;A\;\times e^{\left(\frac{-\Delta t}{\tau }\right)}$$

where *A* is the scaling factor, *τ* is the time constant, and Δ*W* is a change in the synaptic weight. The values of *A* and *τ* are mentioned in the inset of each figure. The value for changing the synaptic weight (Δ*G*/*G*0 or Δ*W*) was slightly low because of the imprecise pre‐/post‐spike timing window. However, this could be improved by modifying the amplitude and the time spacing between the individual pulses and providing smaller amplitude spikes with a larger number of repetition pairs.^[^ ^40^ ^]^ The fitted values and time scale of the STDP learning rules were similar to those of biological synapses and other artificial synaptic devices.^[^ ^41^ ^]^ These experimental results suggested that the MemOSC worked in a similar way to an artificial synaptic device and would be a potential candidate for neuromorphic computing applications. The RS and synaptic performance of the fabricated MemOSC device and the existing organic memory device are compared in Table S3, Supporting Information. In addition, correlations between the OSC parameters and switching cycles of the MemOSC device were studied. The effect of memristive channel formation on the initial values of PCE, *V*_oc_, *J*_sc_, and FF are plotted in Figure 4i. Although the photoconversion efficiency of the device was reduced after several switching cycles, 84% of its initial efficiency was retained after 100 consecutive cycles. These results confirmed that the proposed MemOSC device could use an identical stack for simultaneous energy harvesting, nonvolatile memory, and neuromorphic computing applications. Finally, the switching mechanism of the MemOSC device was investigated by examining the conductive atomic force microscopy (C‐AFM), as shown in Figure S13a,b, Supporting Information, and accordingly, a plausible RS mechanism is depicted in Figure S13c,d, Supporting Information. These results confirmed that the filamentary type RS mechanism was dominated in the MemOSC device.

## 3 Conclusion

In this study, we demonstrated the successful synthesis of MXene hybrids, which were then implemented to fabricate multifunctional FTCEs. One of the optimized FTCEs (Zn@MAM) exhibited high transmittance (84%), a low sheet resistance (9.7 Ω sq^−1^), and passed the 2000 cyclic bending test without any severe damage. In addition, the OSC device optimized with the Zn@MAM electrode exhibited a PCE of 13.86%, which was the highest recorded value among the OSCs that employed MXene‐based electrodes. The results of this study provide a potential method for obtaining high‐performance, long‐term‐stable flexible large‐area OSCs with excellent mechanical properties using multilayer hybrid MXene electrodes. Furthermore, the same material stack was used to investigate the simultaneous achievements of solar energy harvesting, nonvolatile memory, and synaptic learning functionalities. Interestingly, the MemOSC worked at very low switching voltages (0.60 and −0.33 V) and exhibited a high on/off ratio (10^3^), stable endurance cycles (4 × 10^3^), and excellent memory retention (>10^4^ s) without any detectable memory loss. Moreover, the proposed MemOSC device mimicked the basic and advanced synaptic learning properties, rendering it suitable for brain‐inspired computing applications. We also demonstrated its stable resistive switching characteristics over hundreds of switching cycles without any serious damage to the PCE and other photovoltaic parameters. Ultimately, this study demonstrated that MXene‐based electrodes perform well in flexible OSCs and have great potential for fabricating electrically readable nonvolatile memory and artificial synaptic devices for simultaneous energy harvesting, memory, and neuromorphic computing applications towards future multifunctional integrated device applications.

## 4 Experimental Section

### Chemicals

Poly[[4,8‐bis [5‐(2‐ethylhexyl)‐4‐fluoro‐2‐thienyl]‐benzo[1,2‐b:4,5‐b′]dithiophene‐2,6‐diyl]‐2,5‐thiophenediyl[5,7‐bis(2‐ethylhexyl)‐4,8‐dioxo‐4H,8H‐benzo[1,2‐c:4,5 c′]dithiophene‐1,3‐diyl]‐2,5‐thiophenediyl] (PM6), (2,2ʹ‐((2Z,20Z)‐((12,13‐bis(2‐ethylhexyl)‐3,9‐diundecyl‐12,13‐dihydro‐[1,2,5]thiadiazolo[3,4‐e]thieno[2,ʺ30ʹ:4ʹ,50]thieno[20,30:4,5]pyrrolo[3,2 g]thieno[20,30:4,5]thieno[3,2‐b]‐indole‐2,10‐diyl)bis(methanylylidene))bis(5,6‐difluoro‐3‐oxo‐2,3‐dihydro‐1H‐indene‐2,1‐diylidene))dimalononitrile) (Y6), [6,6]‐Phenyl C71 butyric acid methyl ester (PC_71_BM), chloroform (CF) and chloronaphthalene were purchased from Sigma Aldrich. The Ti_3_AlC_2_ powder was supplied by XF NANO, Jiangsu, China.

### Preparation of MXene Nanosheets

The Ti_3_AlC_2_ was selectively etched to obtain the Ti_3_C_2_T*_x_* MXene nanosheets_._ In detail, 1 g of Ti_3_AlC_2_ was added and dispersed in 20 mL of 49% hydrofluoric acid (HF) with a sufficient time interval to prevent H_2_ bubbling_._ Then, the mixture was stirred for 18 h at 50°C, and the precipitate was washed with deionized water and ethanol to remove residual HF or impurities. The pH of the solution was adjusted to neutral. Finally, the MXene powder was collected and vacuum dried for 24 h at 80 °C.

### Delamination of MXene Nanosheets

For the delamination, 0.1 g of MXene powder was added to 5 mL of DMSO and magnetically stirred for 24 h at room temperature (RT). DMSO‐intercalated MXene was centrifuged at 3500 rpm for 10 min, and the supernatant was decanted. The remaining product was diluted with distilled water (150 mL) and subjected to delamination using probe sonication (Ultrasonic Homogenizer, KUS‐650, KBT Co., Ltd.) for 6 h under N_2_ bubbling. Subsequently, the resultant delaminated MXene (DMX) solution was centrifuged at 3000 rpm for 30 min, and the supernatant was stored in the glove box for further use.

### Preparation of MXene Hybrids

Here, 1 mL 1 M aqueous solutions of AgNO_3_, NiCl_2_.6H_2_O, and ZnCl_2_.H_2_O were added into the original DMX solutions and were sonicated for 15 min to prepare MXene hybrids. The obtained solutions of MXene hybrid composites were centrifuged at 8000 rpm, and supernatants were stored in vacuum desiccators at RT.

### Fabrication of the Electrodes

Initially, the PEN flexible substrates were immersed sequentially in detergent solution, deionized water, and IPA sonication baths for 10 min each. Substrates were bowled with nitrogen and the surface was treated with oxygen plasma for 2 min. DMX solution was spin‐coated on the substrate at 800, 1200, and 1500 rpm for 5, 10, and 30 s, respectively. MXene‐coated substrates were vacuum dried at 80 °C for 30 min. Furthermore, a thin 6‐nm layer of Ag was sputter deposited on MXene‐coated substrate at 5 mTorr. Finally, the NPs modified MXene were spin‐coated on Ag at 3000 rpm for 40 s and vacuum annealed (vacuum oven, Sam Heung Energy, VDO‐PK‐S1,) at 60 °C for 30 min. Thin films were stored in a nitrogen glove box at RT.

### Solar Cell Fabrication

A 40‐nm thin layer of ZnO was sputter coated on the fabricated multilayer electrode. For the photoactive layer, 16 mg mL^−1^ PM6:Y6 (1:1.2) and 16 mg mL^−1^ PM6:Y6: PC_71_BM (1:1.2) were dissolved in chloroform and 1‐chloronaphthalene (0.5%). The organic blend solution was stirred at 50 °C for 3 h. Then, the solution was spin‐coated at 3000 rpm for 40 s and annealed at 90 °C for 10 min. Finally, the 10 nm thin MoO_3_ and 120 nm thick Ag electrodes were thermally deposited by using a shadow mask under high pressure of ≈10^−7^ Pa.

### Characterization

XRD patterns were recorded using an X‐Ray diffractometer (Rigaku, Smart Lab). The sheet resistance of different electrodes was measured by a probe tester (CMT‐SR2000N, AIT Co. Ltd.), and transmittance spectra were recorded with a UV‐Vis spectrometer (Lambda‐35, PerkinElmer). The surface morphology of electrodes after organic layer deposition was recorded using the AFM (Park XE‐100) in the tapping mode. Current profiles at LRS and HRS were measured by the C‐AFM technique (Park system XE‐100). XPS measurements of MXene and its composites were investigated using an X‐ray photoelectron spectroscope (X‐tool, ULVAC‐PHI). The photovoltaic characteristics in the forward bias were achieved by solar cell *I–V* test system (K3000, McScience) through Keithley 2400 source measurement unit under AM 1.5 (100 mW cm^−2^). EQE spectra of OSCs were measured by the solar cell IPCE measurement system (K3100, McScience). The electrical and synaptic properties were measured using ArC ONE (UK) and Keithley 4200A instruments.

## Conflict of Interest

The authors declare no conflict of interest.

## Supporting information

Supporting Information

## Data Availability

The data that support the findings of this study are available from the corresponding author upon reasonable request.
