# Supplementary material for: Flexible Memristive Organic Solar Cell Using Multilayer 2D Titanium Carbide MXene Electrodes
Source: Adv Sci (Weinh). 2023 May 3;10(19):2300433. doi: 10.1002/advs.202300433 (PMC10323661; doi:10.1002/advs.202300433)
Supplement: Supplementary file 1 — Supporting Information [file ADVS-10-2300433-s001.pdf]

ADVANCED SCIENCE
Open Access

Supporting Information

for *Adv. Sci.*, DOI 10.1002/advs.202300433

Flexible Memristive Organic Solar Cell Using Multilayer 2D Titanium Carbide MXene Electrodes

*Kiran A. Nirmal, Wanqi Ren, Atul C. Khot, Dae Yun Kang, Tukaram D. Dongale and Tae Geun Kim**

Supporting Information

Flexible Memristive Organic Solar Cell (MemOSC) using Multilayer 2D Titanium Carbide MXene Electrodes

*Kiran A. Nirmal‡, Wanqi Ren‡, Atul C. Khot, Dae Yun Kang, Tukaram D. Dongale and Tae Geun Kim**

K. A. Nirmal, W. Ren, A. C. Khot, Dr. D. Y. Kang, Prof. T. G. Kim
School of Electrical Engineering, Korea University, Anam-ro 145, Seongbuk-gu, Seoul
E-mail: tgkim1@korea.ac.kr

Dr. T. D. Dongale
Computational Electronics and Nanoscience Research Laboratory, School of Nanoscience and Biotechnology, Shivaji University, Kolhapur 416004, India

‡These authors have equally contributed to this work

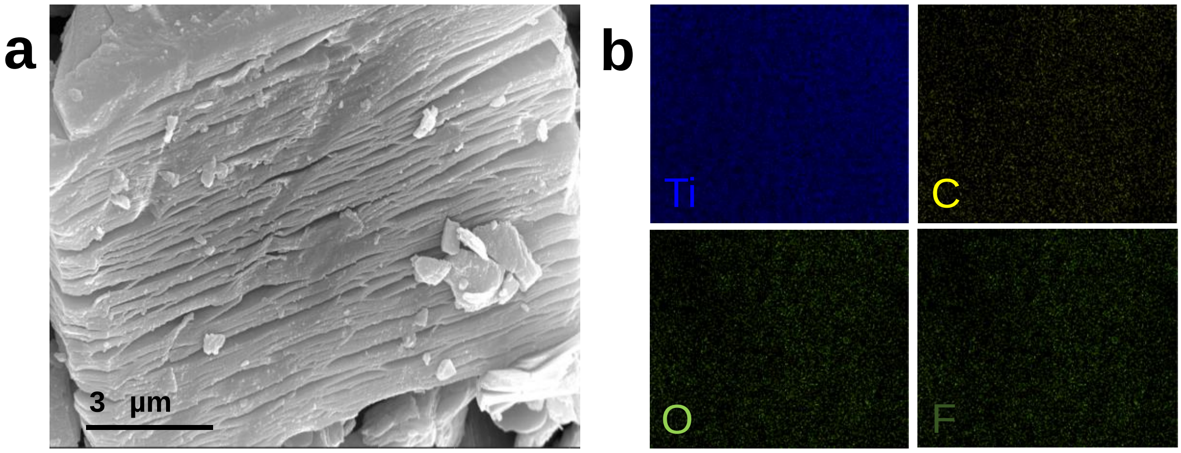

Figure S1. (a) SEM image of $Ti_3C_2T_x$ MXene and its corresponding (b) EDX mapping results.

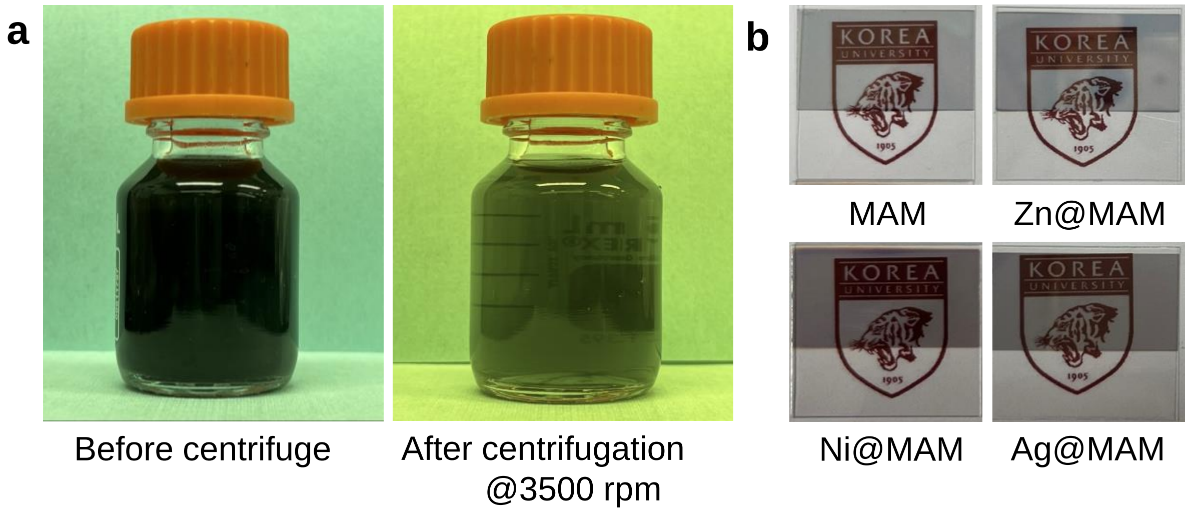

Figure S2. (a) Ultrasonically delaminated colloidal MXene solution and colloidal solution after centrifugation at 3500 rpm. (b) Photographs of fabricated transparent multilayer MXene-based electrodes.

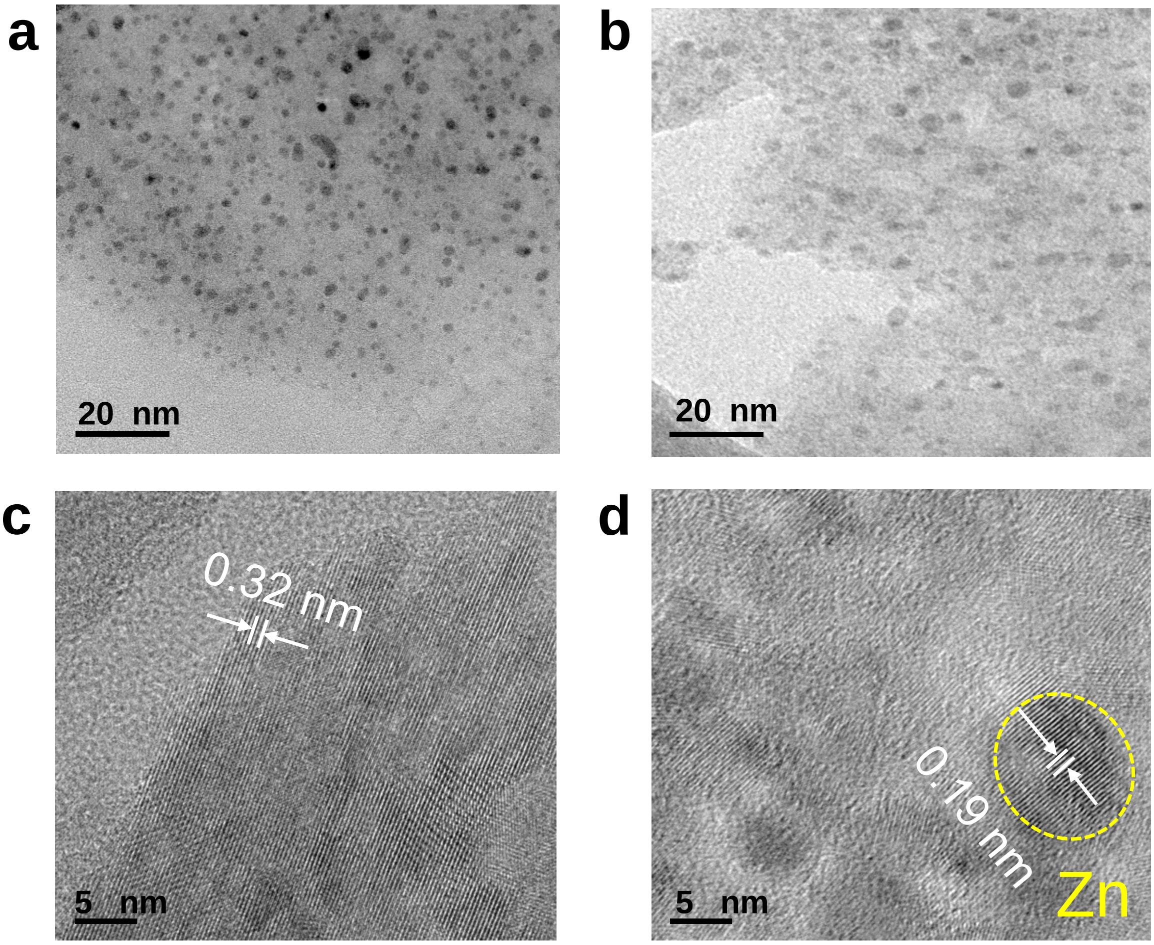

Figure S3. HRTEM images of (a) Ag@$Ti_3C_2T_x$ and (b) Ni@$Ti_3C_2T_x$ colloidal solutions. HRTEM images of pristine (c) $Ti_3C_2T_x$ MXene and (d) Zn@$Ti_3C_2T_x$ with a lattice spacing of 0.32 and 0.19 nm, respectively.

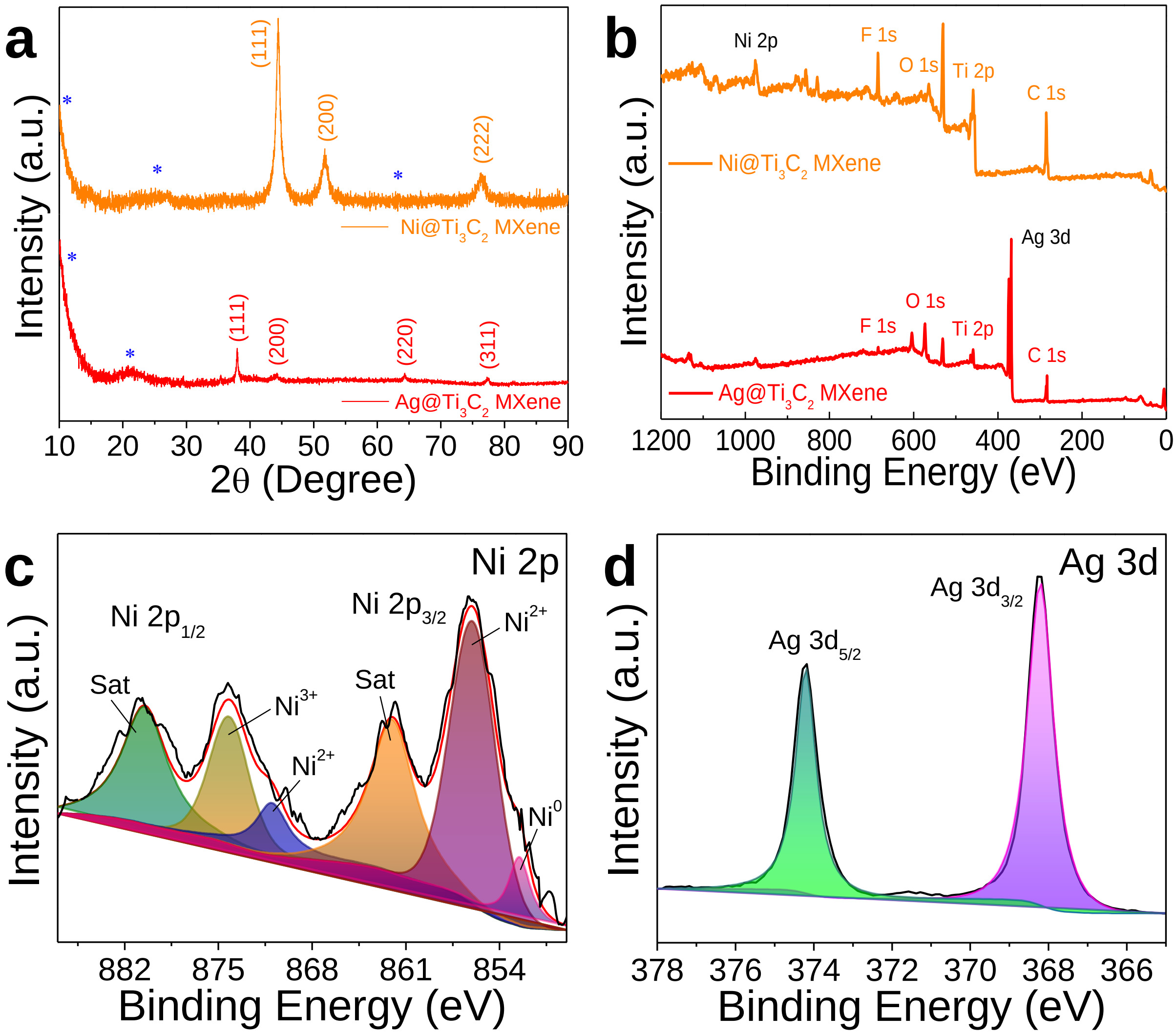

Figure S4. (a) XRD and (b) XPS survey spectra of $Ag@Ti_3C_2T_x$ and $Ni@Ti_3C_2T_x$. High-resolution spectra of (c) Ni 2p and (d) Ag 3d core levels.

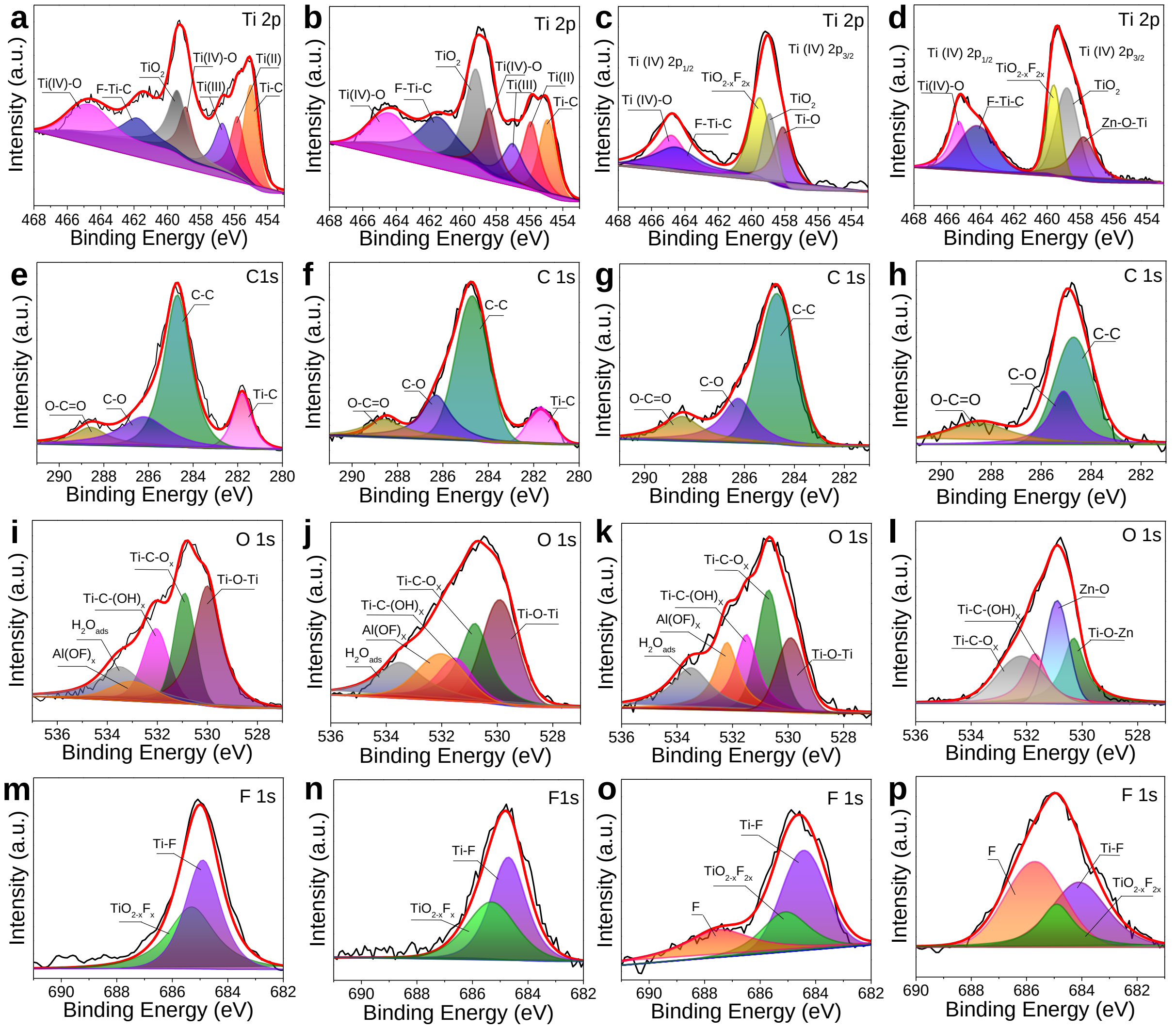

Figure S5. High-resolution deconvoluted XPS spectra of (a–d) Ti, (e-h) C 1s, (i-l) O 1s, (m-p) F 1s of pristine $Ti_3C_2T_x$ MXene, Ni@$Ti_3C_2T_x$, Ag@$Ti_3C_2T_x$ and Zn@$Ti_3C_2T_x$ MXene, respectively.

Gaussian–Lorentzian fitting of the XPS Ti 2p spectrum (**Figure S5a**) of DMX suggested the presence of the titanium in doublets (i.e., Ti $2p_{1/2}$-Ti $2p_{3/2}$). The major peaks of Ti $2p_{1/2}$ are fitted for Ti-C, Ti (II), and Ti (III), corresponding to 455, 455.8, and 456.7 eV, respectively. The minor peaks of Ti $2p_{3/2}$ are observed at 461.8 and 464.7 eV, which are assigned to F-Ti-C and Ti (IV)-O, respectively. Similar bonding is observed in the case of Ni@MXene (**Figure S5b**) with slight changes in the values of the binding energy, as mentioned in **Table S2**. The Ag@MXene hybrid is formed by a self-reduction process via low valence Ti species that act as electron donors (i.e., Ti (II) and Ti (III)) Meanwhile, the low oxidation states are terminated to Ti (IV), diminishing the presence of Ti (II) and Ti (III).

Thus, only two peaks of Ag@MXene match well with the pristine delaminated MXene Ti 2p peak (**Figure S5c**). Similarly, the Ti 2p spectrum of Zn@MXene is deconvoluted into two major groups, as shown in **Figure S5d**. One is with Ti atoms linked to termination groups (Ti (IV)-O, F–Ti–C) and the other is Ti atoms linked to O (Zn-O-Ti, TiO_2-xF_{2x}, and TiO_2). The Zn-O-Ti peak is strong as well as peak position of Ti $2p_{3/2}$ is higher (459.42 eV) than the delaminated MXene (455.1 eV). This result signifies $Ti_3C_2T_x$ MXene is hybridized with Zn on its surface.

The high-resolution XPS spectra of C 1s for pristine MXene appear in four bonds, as shown in **Figure S5e**. The binding energy peaked at 281.8 eV, which corresponds to the carbide component Ti-C. The other three peaks are associated with graphitic C-C (284.7 eV) and surface contaminations of CO (286.2 eV) and COO (288.6 eV). C 1s peak of Ni@MXene shows analogous behavior with the C 1s peak of pristine MXene with a less intense Ti-C peak and a small change in the binding energy (**Figure S5f**). C-C peak appeared because of the selective dissolution of Ti during chemical etching. However, similar to Ti 2p XPS spectra, the tiny peak of Ti-C completely disappears in Ag@MXene (**Figure S5g**) and Zn@MXene (**Figure S5h**) indicating the hybridization of MXene with relevant metal ions.

Moreover, O 1s XPS spectra of delaminated MXene (**Figure S5i**), Ni@MXene (**Figure S5j),** and Ag@MXene (**Figure S5k**) can be deconvoluted into five peaks. Two peaks centered at low binding energy, and these are associated with metal-oxygen bonds and successive committed to hydroxyl groups (**Figure S5i**). The peak centered at 533.5 eV may arise due to adsorbed water at the surface. However, major peaks in O 1s of Zn@MXene are located at 530.9 and 530.3 eV, representing Zn-O and Ti-Zn-O, respectively. The major component of F 1s is associated with the C-Ti-F_x bond, and the sample contains fractions of $TiO_{2-x}F_{2x}$ and F termination (**Figure S5m-p**). These results suggested that the MXene surface randomly ends with F, OH, and O.

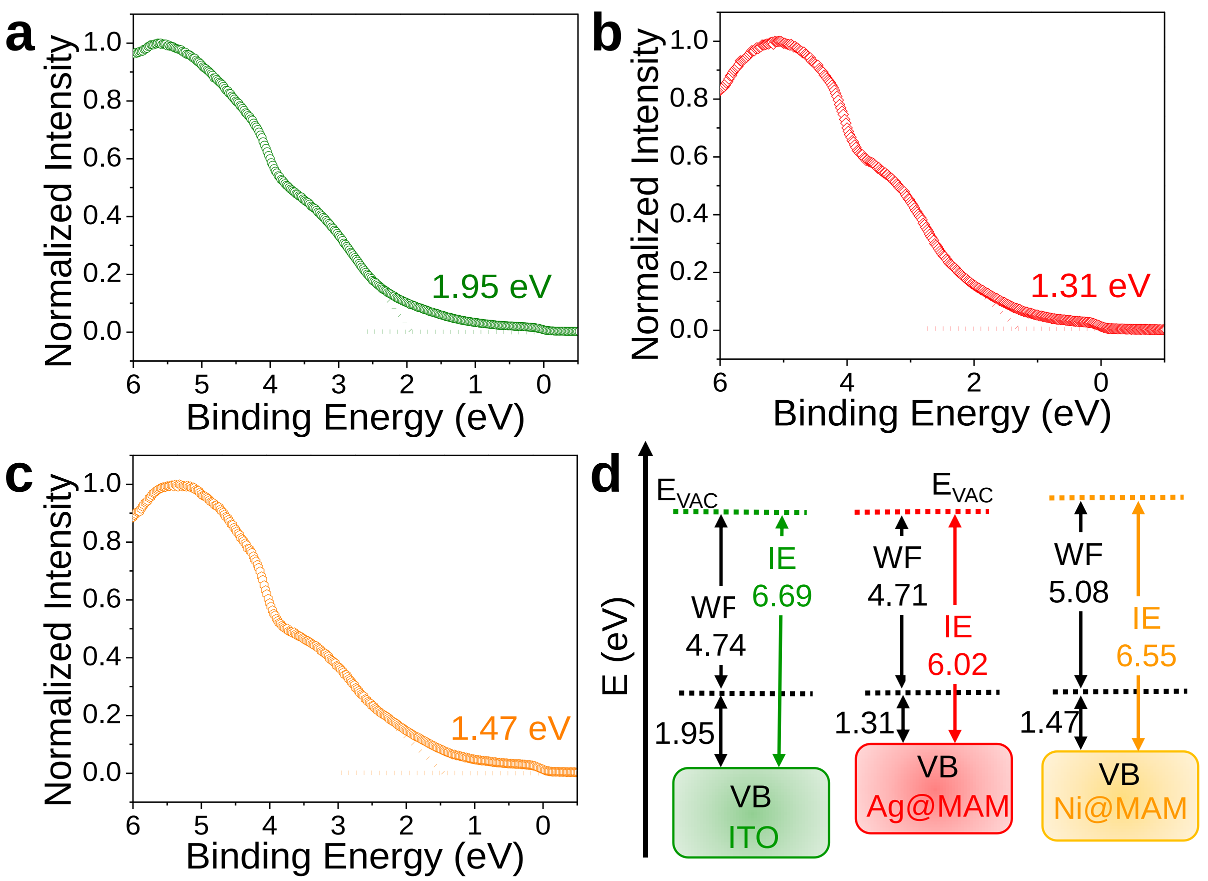

Figure S6. UPS spectra were measured with a photon energy of 21.22 eV on (a) ITO, (b) Ag@MAM, and (c) Ni@MAM electrodes in the valence band region, respectively. (d) Energy level diagrams of ITO, Ag@MAM, and Ni@MAM electrodes.

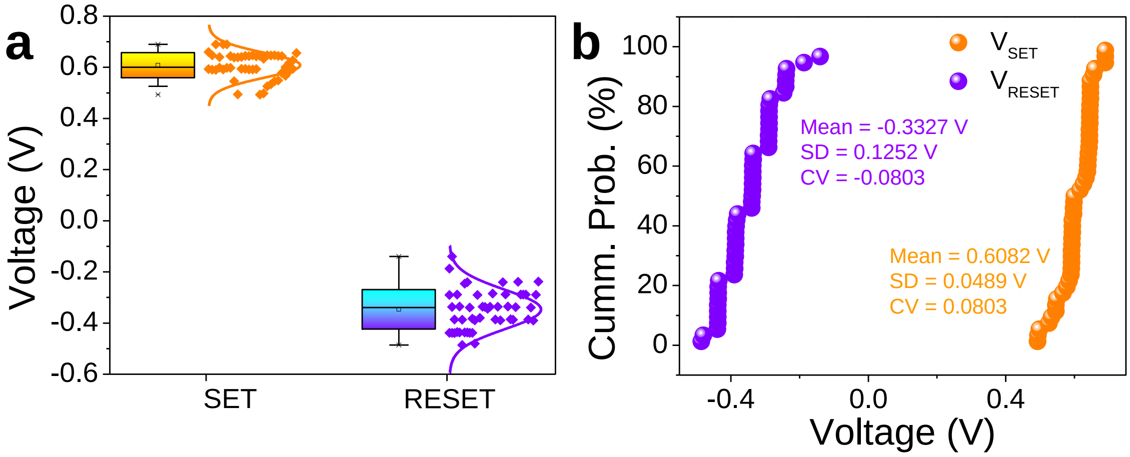

Figure S7. (a) Box plot distribution and (b) cumulative probability of switching voltages.

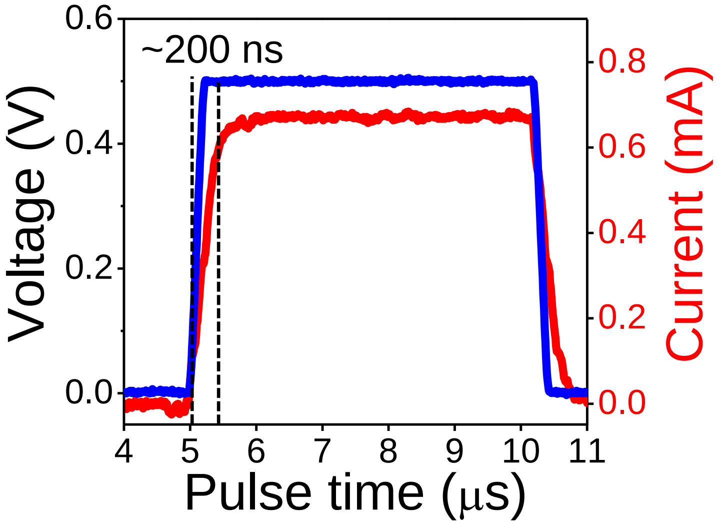

Figure S8. Switching speed characteristics of the MemOSC device.

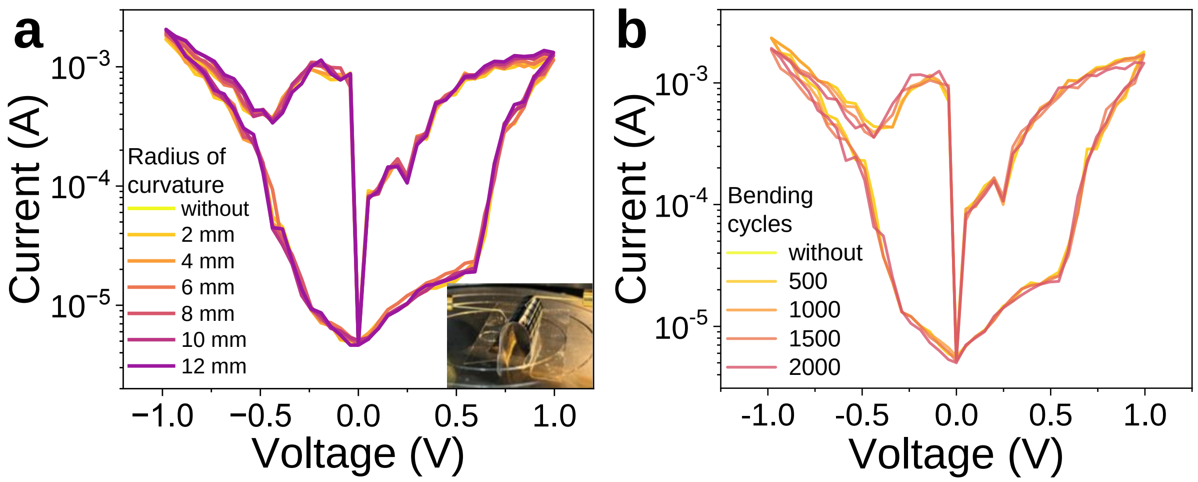

Figure S9. *I-V* characteristics of the MemOSC device with respect to (a) radius of curvature and (b) bending cycles. Inset shows the device under measurement.

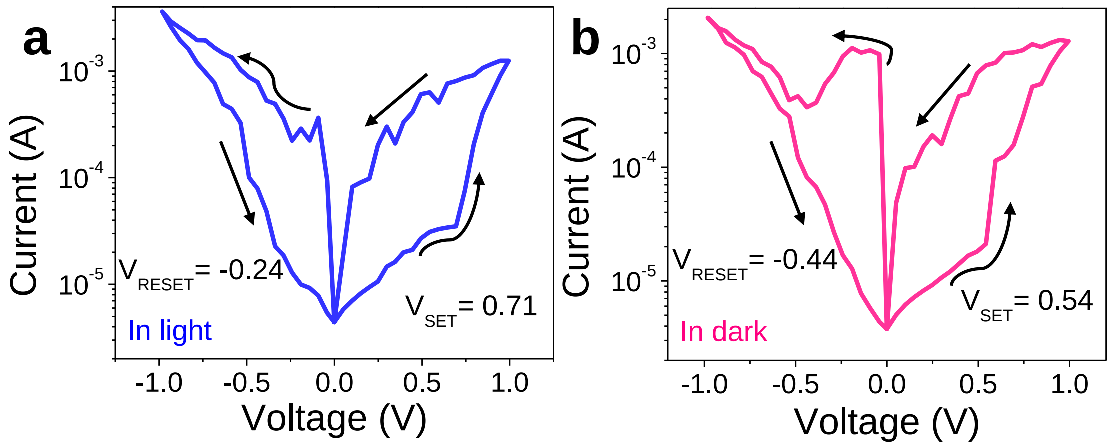

Figure S10. *I-V* characteristics of the device measured under (a) visible light and (b) dark environment. V_{SET} = SET voltage, V_{RESET} = RESET voltage.

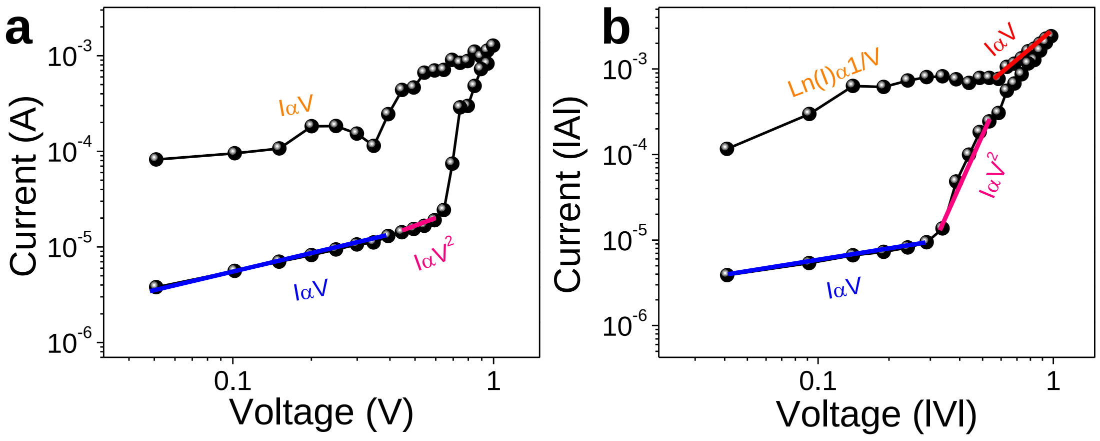

Figure S11. Log (*I*) vs Log (*V*) characteristics of MemOSC.

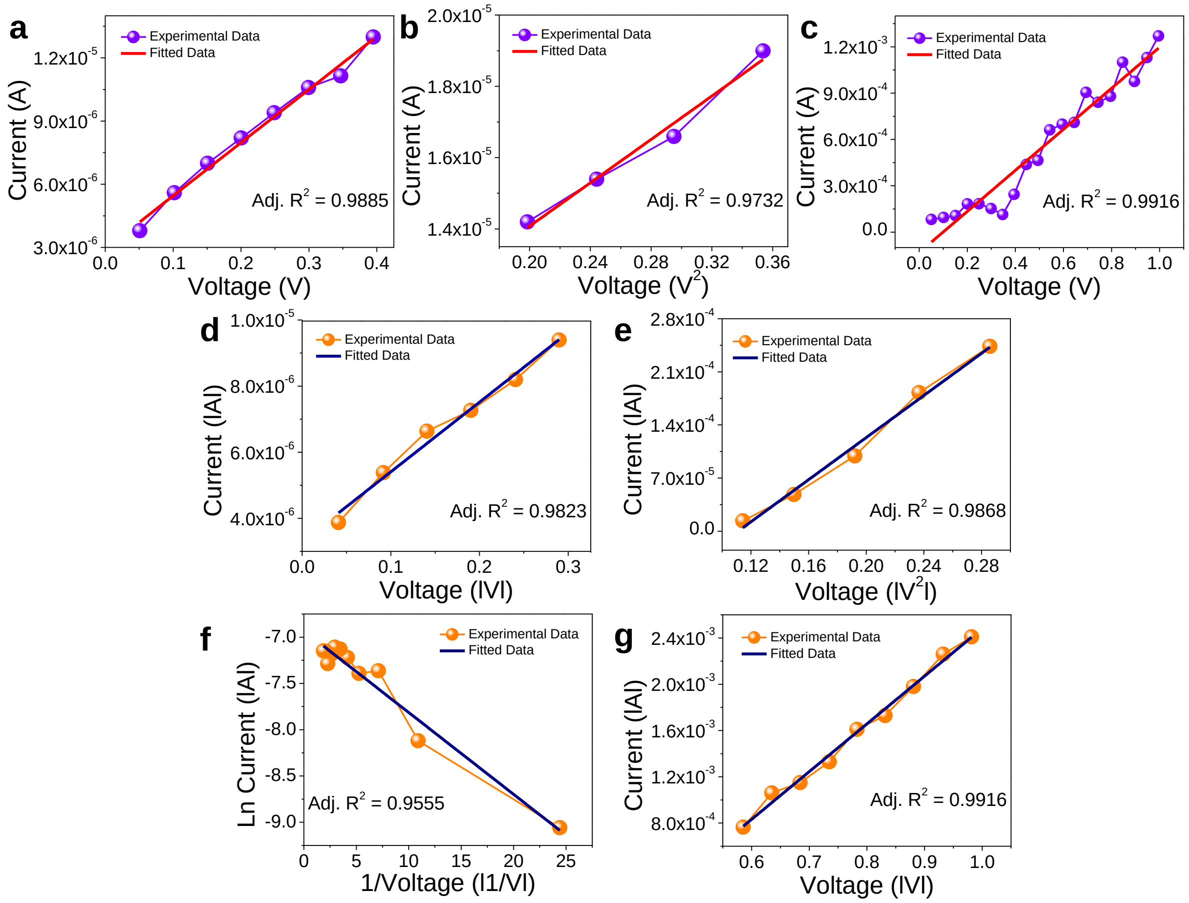

Figure S12. Charge transport fitting results for MemOSC device. Positive bias charge transport results in (a) ohmic conduction fitting the low voltage (HRS), (b) Child's square law fitting the high voltage region (HRS), and (c) entire LRS fitting well to the ohmic conduction. Negative bias charge transport results: low voltage fits for (d) ohmic conduction fits the low voltage (HRS), (e) Child's square law fits the high voltage region (HRS), and entire LRS fits well to (f) trap assisted tunneling and (g) ohmic conduction.

The *I-V* curves were plotted on a double logarithmic scale (**Figure S8 a and b**) and fitted using different charge transport models (**Figure S9 a-g**) to verify the type of charge transport mechanism. The measured *I-V* data are fitted to different charge transport models at different voltage regions. The signature of space charge limited conduction (SCLC) and trap-assisted tunneling (TAT) conduction mechanisms is observed in the device. Ohmic transport is dominant in the low voltage region of HRS due to the low density of injected carriers than the thermally generated carrier density. On the other hand, the Child's square law is dominant in high-voltage regions of HRS. Thereafter, a sudden increase in current was observed due to

the formation of conductive filament(s). Meanwhile, the LRS of the positive bias region was dominated by ohmic conduction. In contrast, the low voltage region of negatively biased LRS follows the TAT conduction mechanism, and the high voltage region obeys the ohmic law.

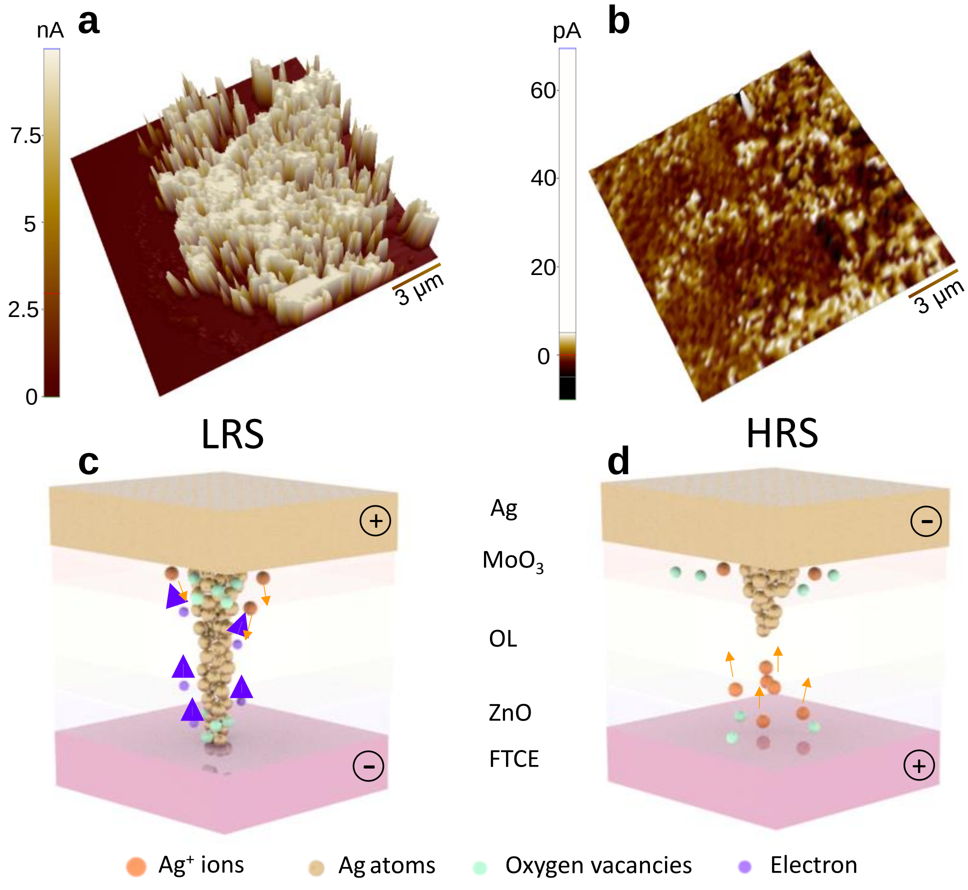

Figure S13. C-AFM image of the (a) LRS and (b) HRS. The proposed switching mechanism of the MemOSC device during (c) LRS and (d) HRS.

Figures S13a and S13b display spatial current maps (measured using C-AFM) at LRS and HRS of the MemOSC device, indicating the generation and dissolution of conductive filaments at each state. These results directly indicate that the filamentary RS mechanism dominates the switching operation of the device. Based on these results, a filamentary switching process that could be applied to the present device is schematically illustrated in **Figures S13c and S13d**.

Specifically, in **Figure S13c**, the active Ag metal atoms are oxidized into Ag^+ ions when a positive voltage is applied to TE and Ag^+ ions start to migrate toward BE. Meanwhile, the electrons generated at BE travel toward TE. Under forward bias conditions, precipitation of Ag^+ occurs at many sites and traveling Ag^+ ions are reduced by the combination of moving electrons. The continuous growth of the Ag filament then bridges the electrodes, creating a

highly conductive pathway of free carriers, turning the device into the LRS, as depicted in **Figure S13c**. Moreover, oxygen vacancies induced from ZnO and MoO_3 can assist in the formation of a strong conductive filament.

Conversely, under reverse bias conditions, the filaments are ruptured via Joule heating. In this case, TAT and ohmic conduction are found to be dominant in the LRS region because the trapped electrons are extracted with increased voltages. Finally, the conduction path changes from SCLC to ohmic type again after the LRS is switched to HRS because Ag^+ ions collocated at TE are reduced to Ag, as shown in **Figure S13d**. The proposed mechanism is in agreement with the results reported in the literature.[1,2] For example, Patil et al. reported that device operation in an organic bulk heterojunction is typically governed by an ion migration (Ag^+)-assisted filamentary resistive switching mechanism.[1] In addition, Krishnan et al. reported a similar operational mechanism with Ag^+ and ClO_4^- ionic species as charge carriers in Ag salt-incorporated polyethylene oxide.[2]

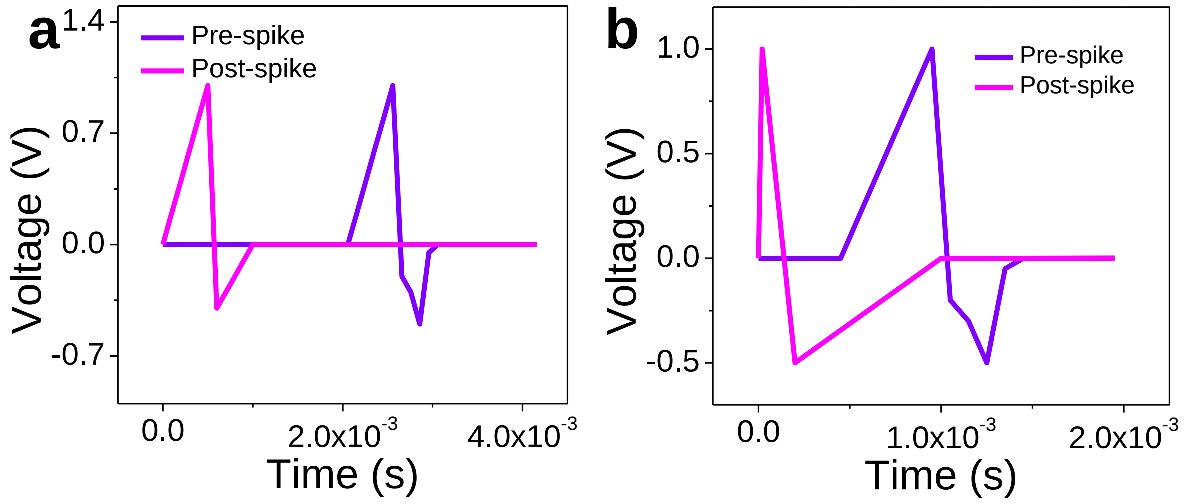

Figure S14. STDP-based pulse schemes to measure (a) ASH and (b) ASAH learning rules.

Table S1. Role of MXene in different organic solar cells.

No.	Chemical structure	Work function	Use	PCE	References
1	$Ti_3C_2T_x$	4.08 - 4.95	ETL	9.06 %	*[3]*
2	$Ti_3C_2T_x$	5	HTL	10.53 %	[4]
3	$Ti_3C_2T_x$/Ag	5.28	FTCE	8.3 %	*[5]*
4	$Ti_3C_2T_x$	4.36	TCE	13.6 %	*[6]*
5	$Ti_3C_2T_x$	5.07	TCE	1.12 %	*[7]*
6	$Ti_3C_2T_x$	-	Additive in active layer	16.25 %	*[8]*
7	$Ti_3C_2T_x$ /PEDOT:PSS	4.9	Anode interfacial layer	17.26 %	*[9]*

Table S2. Summary of XPS quantitative data.

Type	Region	BE (eV)	FWHM (eV)	Area under curve	Assigned to
DMXene	Ti 2p	455.0	1	3391	Ti-C
		455.8	1	2591	Ti (II)
		456.7	1.2	2350	Ti (III)
		458.9	1	2400	Ti (IV)-O
		459.4	1.4	4070	TiO_2
		461.8	2.2	2390	F-Ti-C
		464.7	2.8	2690	Ti (IV)-O
	C 1s	281.8	1	1465	Ti-C
		284.7	1.4	5665	C-C
		286.2	2.5	2165	C-O
		288.6	1.7	995	O-C=O
	O 1s	530	1.3	6317	Ti-O-Ti
		530.9	1	3817	Ti-C-O_x
		532.1	1.2	3017	Ti-C-$(OH)_x$
		533	2	1136	Al $(OF)_x$
		533.5	2	3017	H_2O_{ads}
	F 1s	684.9	1.5	3507	Ti-F
		685.3	2	2977	$TiO_{2-x}F_{2x}$
Ni@MXene	Ni 2p	856	4	3600	Ni^{2+}
		862.1	4	2300	Satellite
		871.1	3	669	Ni^{2+}
		874.2	3.5	1450	Ni^{3+}
		880.5	4	1800	Satellite
	Ti 2p	454.9	1.2	2407	Ti-C
		455.9	1.2	2487	Ti (II)
		457	1.5	2137	Ti (III)
		458.4	1.1	2137	Ti (IV)-O
		459.2	1.5	4037	TiO_2
		461.5	2.6	2937	F-Ti-C
		464.4	2.7	2337	Ti (IV)-O
	C 1s	281.7	1.2	996	Ti-C
		284.7	1.6	5221	C-C
		286.1	1.5	1421	C-O
		288.5	1.4	604	O-C=O
	O 1s	529.9	1.5	5034	Ti-O-Ti
		530.8	1.3	4033	Ti-C-O_x
		531.5	1.5	2136	Ti-C-$(OH)_x$
		532	2	2868	Al $(OF)_x$
		533.5	2	3033	H_2O_{ads}
	F 1s	684.7	1.5	2858	Ti-F
		685.3	2.1	2189	$TiO_{2-x}F_{2x}$
	Ag 3d	368.2	0.8	52588	Ag $3d_{5/2}$
		374.2	0.5	33788	Ag $3d_{3/2}$

Ag@MXene	Ti 2p	458.1	1.3	2389	Ti-O
		458.9	1	1789	TiO_2
		459.5	1.5	3289	$TiO_{2-x}F_{2x}$
		464.5	3	2289	F-Ti-C
		464.8	2	2389	Ti (IV)-O
	C 1s	284.7	1.7	5108	C-C
		286.3	1.5	1338	C-O
		288.5	1.7	1138	O-C=O
	O 1s	529.9	1.2	2862	Ti-O-Ti
		530.7	1	4051	Ti-C-O_x
		531.5	1	3271	Ti-C-$(OH)_x$
		532.2	1	3071	Al $(OF)_x$
		533.5	1.7	3171	H_2O_{ads}
	F 1s	684.4	1.9	1159	Ti-F
		685.1	2	529	$TiO_{2-x}F_{2x}$
		687.5	3	729	F
Zn@MXene	Zn 2p	1022.2	1.9	75940	Zn $2p_{3/2}$
		1045.6	1.5	26240	Zn $2p_{1/2}$
	Ti 2p	457.8	1.7	4286	Zn-O-Ti
		458.8	1.6	6886	TiO_2
		459.6	1	4286	$TiO_{2-x}F_{2x}$
		464.2	2.5	5386	F-Ti-C
		465.3	1.2	3386	Ti (IV)-O
	C 1s	284.7	1.8	1443	C-C
		285.1	1.3	743	C-O
		288.5	3.2	743	O-C=O
	O 1s	530.3	1	5356	Ti-O-Zn
		530.9	1	5857	Zn-O
		531.7	1	4056	Ti-C-$(OH)_x$
		532.2	2	5356	Ti-C-O_x
	F 1s	684.1	2.4	438	Ti-F
		684.9	1.5	245	$TiO_{2-x}F_{2x}$
		685.7	2.5	568	F

Table S3. Performance comparison of the fabricated MemOSC device and existing organic memory devices.

Device structure	V_{SET}, V_{RESET} (V)	Endurance cycles (#)	Retention (s)	ON/OFF ratio	Synaptic Learning	Reference
Ag/ZnO/P3HT-PCBM/ITO	+1.9, -2	500	10^4	10^5	-	[1]
Ta/EV(ClO_4)$_2$/BTPA-F/Pt	±1	-	-	-	P/D, PPF, PTP, SRDP, STDP	[10]
Ag/ LCP/ITO	±5	100	3×10^3	10^2	-	[11]
Ag/Nafion/Au	±1.5	105	-	10^3	-	[12]
Au/polyimide/Au	+0.6, -0.8	100	10^4	10^4	-	[13]
Al/eCPFs/ITO	+2, -3	400	10^3	10^4	-	[14]
Al/COF-Azu/ITO	+1.95, -0.5	300	10^3	50	P/D, LTP, LTD	[15]
Al/TFPA-TAPA /ITO	±1	-	-	-	P/D, SRDP PPF, PTP	[16]
Al/TMA: PVP/ITO	+2.75, -2.5	500	10^5	10^2	P/D, PPF, STP, LTP	[17]
Al/NPVCz-1/ ITO	-2.5, 4.5	100	6×10^3	-	-	[18]
Al/pemtacene/PEDOT:PSS/ITO	±1	-	-	-	P/D, PPF/PPD, SDDP, SRDP	[19]
Au/ITS-PMMA/ITO	+17.4, -20	400	2.5×10^3	10^3	-	[20]
Ag/MoO_3/OL/ZnO/FTCE/PEN	+0.6, -0.3	4000	10^4	10^3	P/D, STDP	This work

P/D- potentiation/depression, PPF- Paired-pulse facilitation, PPD- paired-pulse depression, PTP- Post-tetanic potentiation, SRDP- Spike-rate-dependent plasticity, STDP- Spike-time-dependent plasticity, LTP- long term potentiation, LTD- long term depression, STP- short term plasticity, LTP- long term plasticity, SDDP- Spike-duration-dependent plasticity

References

[1] H. Patil, H. Kim, S. Rehman, K.D. Kadam, J. Aziz, M.F. Khan, D.K. Kim, *Nanomater.* **2021**, *11*(2), 359.

[2] K. Krishnan, T. Tsuruoka, C. Mannequin, M. Aono, Adv. Mater. 2016, 28(4), 640-648.

[3] Z. Yu, W. Feng, W. Lu, B. Li, H. Yao, K. Zeng, J. Ouyang, *J. Mater. Chem. A.* **2019**, *7*(18), 11160-11169.

[4] C. Hou, H. Yu, C. Huang, *J. Mater. Chem. C.* **2019**, *7*(37), 11549-11558.

[5] H. Tang, H. Feng, H. Wang, X. Wan, J. Liang, Y. Chen, *ACS Appl. Mater. Interfaces* **2019,** *11*(28), 25330-25337.

[6] L. Qin, J. Jiang, Q. Tao, C. Wang, I. Persson, M. Fahlman, P.O. Persson, L. Hou, J. Rosen, F. Zhang, *J. Mater. Chem. A* **2020**, *8*(11), 5467-5475.

[7] P. Wang, M. Jian, C. Zhang, M. Wu, X. Ling, J. Zhang, B. Wei, L. Yang, *Adv. Mater. Interfaces* **2022**, *9*(3), 2101442.

[8] Y. Zhao, X. Liu, X. Jing, X. Wang, C. Gao, S. Dai, L. Yu, M. Sun, *Solar RRL* **2021**, *5*(4), 2100127.

[9] J. Wang, R. Peng, J. Gao, D. Li, L. Xie, W. Song, X. Zhang, Y. Fu, Z. Ge, *ACS Appl. Mater. Interfaces* **2021**, *13*(38), 45789−45797.

[10] G. Liu, C. Wang, W. Zhang, L. Pan, C. Zhang, X. Yang, F. Fan, Y. Chen, R.W. Li, *Adv. Electron. Mater.* **2016**, (2), 1500298.

[11] H.L. Park, M.H. Kim, S.H. Lee, *Organic Electron*. **2020**, *87*, 105927.

[12] Y. Xu, H. Wang, D. Ye, R. Yang, Y. Huang, X. Miao, *IEEE Electron Device Lett.* **2021**, *43*(1),116-119.

[13] W. Wang, G. Zhou, Y. Wang, B. Yan, B. Sun, S. Duan, Q. Song, *J. Phys. Chem. Lett.* **2022**, *13*(42), 9941-9949.

[14] Y. Tao, H. Liu, H.Y. Kong, T.X. Wang, H. Sun, Y.J. Li, X. Ding, L. Sun, B.H. Han, *Angew. Chemie Int. Ed.* **2022**, *61*(38), 202205796.

[15] Z. Zhao, M.E. El□Khouly, Q. Che, F. Sun, B. Zhang, H. He, Y. Chen, *Angew. Chemie Int. Ed.* **2023**, *62*(7), 202217249.

[16] D. Wu, Q. Zhang, X. Wang, B. Zhang, *Nanoscale* **2023**, *15*, 2726-2733.

[17] A. Betal, J. Bera, S. Sahu, *J. Mater. Chem. C.* **2023** (Non-volatile Memristor-Based Artificial Synaptic Behavior of Redox-Active Organic Composite)

[18] F.L. Ye, C.J. Lu, H.X. Chen, Y.H. Zhang, N.J. Li, L.H. Wang, H. Li, Q.F. Xu, J.M. Lu, *Polym. Chem.* **2014**, *5*(3), 752-760.

[19] X. Luo, J. Ming, J. Gao, J. Zhuang, J. Fu, Z. Ren, H. Ling, L., Xie, *Front. Neurosci.* **2022**, *16*.

[20] S.H. Lee, H.L. Park, M.H. Kim, S. Kang, S.D. Lee, *ACS Appl. Mater. Interfaces* **2019**, *11*(33), 30108-30115.
